# Analgesia effect of a fixed nitrous oxide/oxygen mixture on burn dressing pain: study protocol for a randomized controlled trial

**DOI:** 10.1186/1745-6215-13-67

**Published:** 2012-05-24

**Authors:** Li Yuxiang, Tang Lu, Yu Jianqiang, Dai Xiuying, Zhou Wanfang, Zhang Wannian, Hu Xiaoyan, Xiao Shichu, Ni Wen, Ma Xiuqiang, Wu Yinsheng, Yao Ming, Mu Guoxia, Wang Guangyi, Han Wenjun, Xia Zhaofan, Tang Hongtai, Zhao Jijun

**Affiliations:** 1 Department of Nursing, Changhai Hospital, Second Military Medical University, 168 Changhai Road, Shanghai, 200433, China; 2 Ningxia Medical University, 1160 Sheng Li Street, Yinchuan, 750004, China; 3 Burn and Trauma Centre, Changhai Hospital, Second Military Medical University, 168 Changhai Road, Shanghai, 200433, China; 4 Department of Anesthesiology & ICU, Changhai Hospital, Second Military Medical University, 168 Changhai Road, Shanghai, 200433, China; 5 Medical Statistics Department, Second Military Medical University, 800 Xiangyin Road, Shanghai, 200433, China; 6 Burn and Trauma Centre, General Hospital of Ningxia Medical University, 804 Shengli Street, Yinchuan, 750004, China; 7 Department of Anesthesiology, General Hospital of Jinan Military Command, 25 Shifan Road, Jinan, 250031, China; 8 Department of Phamacology, Second Military Medical University, 800 Xiangyin Road, Shanghai, 200433, China

**Keywords:** Analgesia, Burn procedural pain, Nitrous oxide

## Abstract

**Background:**

Procedural burn pain is the most intense acute pain and most likely type of burn injury pain to be undertreated due to the physician’s fear of the adverse effect of analgesia and lack of anesthetist present. At our institution, in most of the cases, local burn detersion and debridement were performed at the ward level without any analgesics. This article describes a study designed to test the analgesia effect of a fixed nitrous oxide/oxygen mixture on burn dressing pain.

**Methods/design:**

The experiment was carried out in three centers. The patients were given a number from 1 to 240. A randomization list was produced by a statistician according to our preliminary study. Due to the severity of the pain suffered, ethically it was decided to help as many as possible, so patients given the letters A, B or C were treated using a canister with the appropriate letter containing preprepared nitrous oxide/oxygen mixture (NOOM). Those with D were given oxygen only, from an identical-looking canister labeled D. Neither patients, nor doctors, nor nurses, nor data collector knew what was in each canister, thus they were all blind. The nursing officer who implemented the intervention handed the doctors envelopes containing the patients’ name and allocation of A, B, C or D. Thus, patients receiving NOOM or oxygen were in the ratio 3:1. Parameters, including pain severity, blood pressure, heart rate, digital oxygen saturation and the Chinese version of the burn specific pain anxiety scale (C-BSPAS), were taken before, during and after dressing for each group. A video and audio record was taken individually for later communication coding and outcome analysis. Rescue analgesic was recorded.

**Discussion:**

Based on the findings from our previous qualitative study that physician’s reluctance to order narcotic analgesia is due to its adverse effect and from our pilot experiment, this study aims to test the hypothesis that a fixed nitrous oxide/oxygen mixture will promote better burn dressing pain alleviation and outcomes. Analyses will focus on the effects of the experimental intervention on pain severity during dressing (primary outcomes); physiological parameters, C-BSPAS and acceptance of both health care professionals and patients (secondary outcomes). If this model of analgesia for burn pain management implemented by nurses proves successful, it could potentially be implemented widely in hospital and prehospital settings and improve patients’ satisfaction and quality of life.

**Trial registration:**

(Clinical Trials Identifier: CHICTR-TRC11001690).

## Background

Burn injuries are caused by contact with flame, steam, hot fumes, hot liquid, a hot surface, electrical current, or extremely acidic or alkaline chemicals. In China, there are 5 to 10million burn victims each year [1]. Pain is the most common symptom in patients who have experienced burns that require hospital treatment. Severe pain can erode the will to live. Severe burns are among the most painful, devastating, sudden and unpredictable forms of trauma [2]. Acute pain from burns induces anxiety and reluctance by patients to participate in their wound care and rehabilitation, thereby increasing morbidity and extending their hospital stays [3]. Uncontrolled acute pain is also thought to contribute to long-term sensory problems, including chronic pain, paresthesias, allodynia, hyperalgesia, neuropathic pain, phantom skin syndromes and dyesthesias [4-6], associated depression [7], and is correlated with suicidal thoughts at the time of discharge from hospital [8]. The management of burns pain presents a major challenge to the pain practitioner. It is complicated by the fact that burn pain is of nociceptive and/or neuropathic origin and exposes a variety of temporal patterns: constant background pain, intermittent movement-related pain and procedural pain. Patients describe procedural pain as having an intense burning and stinging quality that may continue to a lesser degree but may be accompanied by intermittent sharp pain for minutes to hours after dressing changes. Procedural pain is more severe than background pain and can be excruciating without adequate analgesia. In China, doctors undertake burn wound care including wound washing, dressing and debridement, so they inflict pain and bear the duty to relieve pain for burn patients. Previous studies showed that in some areas of China, burn professionals pay much attention to burn recovery and wound healing. Such experience has advanced the skill of treating burn wounds to international levels, but inadequate attention was paid to the pain management of burns, resulting in untreated or undertreated pain. Our previous qualitative study showed that barriers to effective pain control resided with health care systems and physicians. Due to lack of burn pain management guidelines and assistance of an anesthetist, as well as the fear of adverse effects of analgesia, physicians administrated inadequate analgesia to burn survivors during the dressing and performed oligoanalgesia to children and the elderly by encouraging patients to endure pain [1]. Our recent retrospective study showed that there were 2,436 burn patients discharged from the burns center in a tertiary hospital in China from 1 January 2009 to 30 September 2011. The total hospital cost was ¥192,287,550.40, and the expenditure on medicine was ¥ 56,855,365.48, but only ¥55490.84 was spent on analgesics. The cost of analgesic accounts for 0.0976% of medication in China, whereas in Western countries analgesics account for 50% of the treatment cost. Nearly 60% (58.54%) of patients received oligoanalgesia during their hospitalization. Morphine prescription was zero. Studies have demonstrated multimodal and balanced analgesia were performed in managing burn wounds [9,10].

A China National Knowledge Infrastructure search of the Chinese-language publications from 1994 to 2011 was conducted using the keywords ‘burn pain’ ‘pain management’, and ‘pain control’. This search produced 898 results, of which 21 were found to be relevant to the treatment of burn pain. Seven of the twenty-one were reported using nonpharmacological intervention with three reported by our team using wrist acupuncture to relieve procedure pain for patients undergoing dressing [11]. Of these fourteen pharmacological approaches: seven were studied with a designed randomized control trial, three had placebo control with vitamin C or normal saline and three cases report of clinical administration of intravenous propofol, fantenly and morphine plus midazolam patient-controlled analgesia (PCA) (reported by anesthetists) [12]. Our previous qualitative study showed that pain suffered by burns survivors is untreated and poorly managed. Inadequate treatment of burn pain and inconsistency in practice has been well documented for nearly two decades. Insufficient education, fear of side effects and the economic factors, as well as political reasons and organizational background are major obstacles to progress in burn pain management in burn centers [1]. Our recent study showed that some health care providers in a burn center are also troubled by the dilemma of side-effects of opioids and patients right for freedom from pain. They expressed their apprehensions when facing the unique challenges including the repeated infliction of pain on already traumatized patients with therapeutic procedures. They also stated their willingness to control burn pain as well as their limited knowledge of burn pain management [the result has not published]. Therefore, Chinese burn physicians badly need a safe, effective and economic analgesia implemented by nurses to obtain the optimal control of burns procedure pain. Unfortunately, in China, due to the busy and heavy workload, doctors hardly have the time or interest to explore a burn dressing pain management regime [13].

However, nurses, being around patients’ bedsides and taking care of patients 24 hours a day, are concerned about patients’ comfort. Fortunately, we learned that diluted nitrous oxide is of analgesic effect, so we hypothesized that it may be the most suitable analgesic to meet the need of burn dressing pain management. The analgesia effect of a fixed nitrous oxide/oxygen mixture on burn dressing pain (AEFNOOMBDP) study is a nurse-led, patient-participated, multicenter randomized controlled trial. This article describes the rationale underlying the study, the AEFNOOMBDP study protocol, the design and administration of the study, and the planned analytic approach. Results from the study are expected to be published in 2012.

Nitrous oxide (N_2_0) is an inhaled anesthetic gas possessing analgesic properties at lower concentrations. It is an effective, short-acting analgesic with few side effects and is widely used in obstetrics departments and has also been used prior to a variety of minor surgical procedures [14-20]. Throughout the decade spanning the mid-1980s through 1990s, there was a surge of N_2_0 research in prehospital emergency medical service (EMS) systems across the United States [21-26]. N_2_0 possesses several properties that make it ideal for bedside use in the accident and emergency department. It is easy to administer, requires no intravenous access before its administration, and the patient may control the level of analgesia by removing the gas. It possesses a rapid onset of clinical effect (less than two minutes), and after termination of administration, its effect disappears almost as quickly. It has very few side effects, all of which are self-limited and are generally resolved with termination of exposure to the gas. N_2_0 is widely used in facilities possessing anesthesia services. The apparatus to deliver the gas is inexpensive and portable, and easy for nursing staff to manipulate. But its use in burn dressing pain analgesia alone is rarely reported. We hypothesize that nitrous oxide/oxygen mixture (NOOM) does not completely abolish the pain felt during wound dressing, but rather lessens the intensity and severity of any discomfort that is felt.

## Methods

### Study design

**Figure 1:**
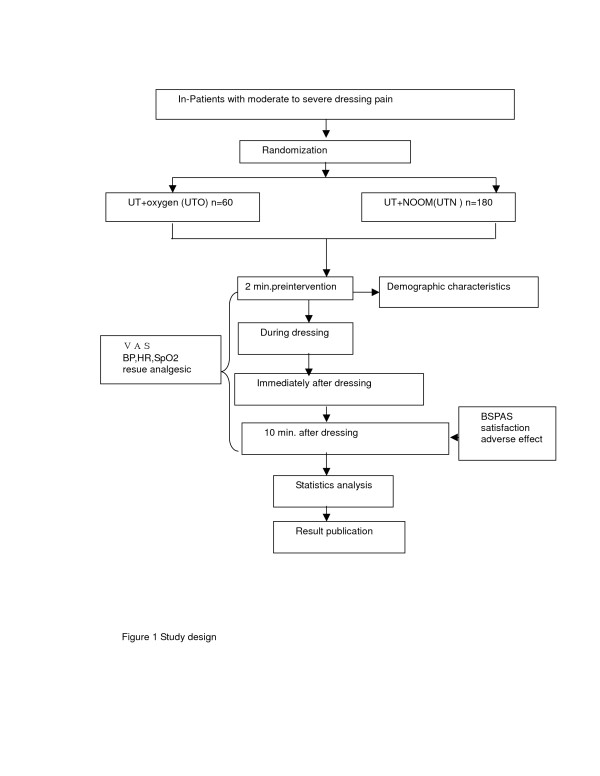
Study design.

The study was designed as a multicenter double-blind randomized controlled trial to compare the analgesic effect of a preprepared NOOM by group UTO (receiving usual pain treatment and oxygen) (n = 60) and group UTN (usual pain treatment and preprepared NOOM) (n = 180) intervention on burn procedure pain in three burns centers under audit for quality control. Research members will be deployed to each study field for measurement taking and evaluating of the results. We choose to randomize individual patients and double-blind because pain threshold varies individually and also due to the placebo effect of the cylinder. The overall study design is depicted in Figure 1.

### Patient eligibility, recruitment, consent, and randomization

Ethical approval was obtained from Shanghai’s Changhai Hospital Ethics Committee (CHEC2011-105). Verbally consenting patients completed a study enrollment pack containing a written consent form and a Chinese versioned burn specific pain anxiety scale (C-BSPAS). Patients eligible for enrollment in the study included all cognitively intact, Chinese speakers aged three to sixty-five from participating burn centers, selected for 1 to 70% of total body surface area (TBSA) burn and who reported moderate to severe burn dressing pain .TBSA was diagnosed by doctors. Moderate pain severity was defined as a score of four or greater (on a scale of zero to ten) for worst pain during dressing. Specific inclusion and exclusion criteria are presented in Table 1.

**Table 1:** Patient inclusion and exclusion criteria

Inclusion Criteria	Exclusion Criteria
Aged 3 to 65, either male or female	Mental disorder, drug dependence and drug abuse
Participants sustained 1-70% of TBSA burns or other injury requiring dressing procedure	Unconscious and unable to express pain by Changhai pain scale (VAS)
Recent worst pain (during dressing) reported to be 4 or higher (on a scale of 0 to 10)	Abdominal distension or suspected bowel obstruction, air embolism, pneumothorax, decompression sickness, epilepsy, chronic obstructive pulmonary disease and acute respiratory infection, pregnancy, severe inhalation injury; pharmaceutical or pathological pulmonary fibrosis; Maxillofacial injuries
The participants are volunteers and written consent obtained	
Burns or injured area not affecting the vital signs and digital pulse oximeter monitoring	Disease involving ear, nose, larynges such as sinuses, middle ear, tympan graft

### Description of experimental and control interventions

Process of allocation concealment; The experiment was carried out in three centers. The patients were given a number from 1 to 240. A randomization list was produced by a statistician according to our preliminary study. Due to the severity of the pain suffered, ethically it was decided to help as many as possible, so patients given the letters A, B or C were treated using a canister with the appropriate letter containing preprepared NOOM. Those with D were given oxygen only, from an identical-looking canister labeled D. Neither patients, nor doctors, nor nurses, nor data collector knew what was in each canister, thus they were all blind. The nursing officer who implemented the intervention handed the doctors envelopes containing the patients’ name and allocation of A, B, C or D. Thus patients receiving NOOM or oxygen were in the ratio 3:1. A sample size of 240 was established and randomized into two groups, for which patients with at least moderate pain severity (worst pain, visual analog score, VAS > = 4 out of 10) during dressing were randomly assigned to receive usual pain treatment plus oxygen (UTO = 60) and usual pain treatment plus preprepared NOOM (UTN, n = 180) intervention two minutes before dressing based on the current clinical practice, respectively. For each group, intervention outcome including pain severity (VAS), blood pressure (BP), heart rate (HR), digital oxygen saturation (SpO_2_) and C-BSPAS were taken two minutes prior to intervention, during dressing, immediately after dressing, and 10 minutes after dressing. Video and audio recordings were taken individually for later communication coding and outcome analyzing. Rescue analgesic was recorded.

Blinding: The double-blind RCT list was held by a researcher. The researcher (a nursing officer), who held the randomizing list was blind too. The list only showed groups A, B, C and D, but did not indicate what treatment these letters stood for. Oxygen intervention was used as a control group. The rules were that the cessation of blinding would take place only after all the trials had been completed.

Indicators present for the expected change in pain between the control and treatment interventions will be examined. The anticipated pain score (VAS) during dressing will be reduced to three to five in the UTN group. The physical parameters such as blood pressure, digital oxygen saturation should be maintained within normal range in the treatment group. The anticipated C-BSPAS will be much lower in the treatment group than that in the control group, and both patients’ and doctors’ satisfaction with the use of the treatment intervention should be obtained after dressing.

### Administration of measures

Demographic characteristics were assessed using administrative records, the screening interview, and the enrollment interview. Pain severity was evaluated with VAS (visual analog scale (zero to ten) on the Changhai pain scale, where zero represents no pain and ten unbearable pain. The scale chosen for pain assessment was categorized as mild (VAS 0 to 4.0), moderate (VAS 4.1 to 6.0) and severe (VAS >6.1)). Physiological parameters: heart rate, blood pressure and digital oxygen saturation were monitored with Infinity Vista XL (Draeger Medial Systems Inc., Danvers, MA, USA). Psychological reactions were measured with the C-BSPAS using five items focusing on dressing-pain-related anxiety. Patient’s satisfaction and the health care professional’s acceptance of diluted nitrous oxide on burn dressing analgesia were audio recorded with an aigo R5518 digital voice recorder (aigo Digital Technology Co. Ltd., Beijing, China). Clinical data, including burns diagnosis, analgesic prescription and hospital cost were obtained via a chart review using a standardized form.

### Sample size determination

A prospective sample size calculation was performed by a senior academic statistician using SAS POWER procedure during the protocol-writing stage. It aimed to determine a target sample size that would provide 90% power for two-tailed testing (at a type-1 error rate of 5%) of each of the measures. According to the result of our preliminary study, a sample size of 12 was targeted as being sufficient to achieve the required effective sample size, but to meet the Chinese Food and Drug Administration standard for safety and feasibility of nursing staff implementing this inhalation analgesic, the targeted sample size of 240 was recommended.

### Data safety and monitoring board (DSMB)

The first patient was enrolled on 14 October 2011. A DSMB was established shortly after the project launch and met several times during the data collection period. Members included two pain management specialists; four burns-trained nursing officers and doctors; and a senior academic statistician who served as the Board's chair.

### Planned analytic approach

In subsequent publications, we will report results pertaining to the analgesic effects of NOOM on burn dressing pain. Primary outcomes will include: 1) pain severity during dressing, measured as the mean of average and worst pain, and 2) burns-dressing-induced anxiety. The physiological parameters and satisfaction of both health care providers and patients are considered as secondary outcomes. Finally, exploratory analyses will assess the interaction between the intervention and age, sex, and racial minority. The primary estimates of the effects of the intervention on each of these outcomes will come from repeated measure analyses, following the intent-to-treat principle.

## Discussion

The AEFNOOMBDP study is a multi-site randomized control trial of a nurse-instructed and patient-controlled intervention designed to reduce pain severity during dressing for burn survivors. Due to special dislike of opioids analgesic in both physicians and patients, burn dressing pain is untreated in China. It is difficult to change attitudes within a short time and most doctors said that burn pain was inevitable and there was no way to control it. Patients screaming out due to dressing pain can be seen. Our previous qualitative study showed that Chinese burn physicians were eager for a safe, effective and economic analgesic regime to control burns dressing pain. Literature suggested that it was safe for a nursing team to provide diluted nitrous oxide analgesia.

A major challenge encountered during implementation of the study was the barrier from the institution and anesthetists due to the confusion of the anesthesic effect of pure nitrous oxide with the analgesic effect of diluted pure nitrous oxide. Seeking approval, we initially recruited ambulatory patients in the outpatient dressing room and took video and audio records to convince the consultants and physicians prior to our preliminary study.

In summary, the AEFNOOMBDP study is in its initial stage and is currently being carried out in one of the three burn centers. Nursing staff were trained to deliver diluted nitrous oxide during burn dressing. Subsequent analyses will focus on the effects of the experimental intervention on dressing pain, anxiety and satisfaction from both doctors and patients.

### Trial status

The study protocol was proposed and carried out in one of the three burn centers.

## Misc

Li Yuxiang, Tang Lu and Yu Jianqiang contributed equally to this work.

## Competing interests

The authors declare that they have no competing interests.

## Authors’ contributions

LYX and TL generated the idea and orchestrated implementation of the protocol. ZJJ managed the project and contributed to the design and statistical analysis plan. ZWF planned the clinical conversation analysis. THT, NW and HXY contributed specialty expertise in pain management. XSC, XZF, WY Sand DXY coordinated implementation of the study at the multicenter site. YJQ and YM coordinated implementation at the academic site. ZWN and MXQ contributed to all aspects of design and analytical planning. WGY, HWJ and MGX served as lead health educators for training nursing staff. All authors read and approved the final version of the manuscript.
